# Supplementary material for: Incidence and predictors of HIV related opportunistic infections after initiation of highly active antiretroviral therapy at Ayder Referral Hospital, Mekelle, Ethiopia: A retrospective single centered cohort study
Source: PLoS One. 2020 Apr 20;15(4):e0229757. doi: 10.1371/journal.pone.0229757 (PMC7170502; doi:10.1371/journal.pone.0229757)
Supplement: S1 File — (DOCX) [file pone.0229757.s001.docx]

Questionnaire For data collection Incidence and predictors of HIV related opportunistic infections after initiation of highly active antiretroviral therapy at Ayder Referral Hospital, Mekelle, Ethiopia. *English Version*

Date of data collection………………

1-Age (Years)………………..

2-Sex A-Male B-Female

3-Marital status A-Single B-Married C-Divorced D-widowed E-separated

4-Employment Status A- Unemployed B- Employed

5-Educational status A- No education B-Primary C-Secondary D- Tertiary

6-Weight (in KG)………………….

7-Height (Meter)……..

8-Residency A-Urban B-Rural

9-Functional status A-working B-Ambulatory C-Bed ridden

10-Hemoglobin level (mg/dl)……………

11-Disclosure status A-Not disclosed B-Disclosed

12-Duration of follow-up ( in months) A-≤34 B-≥35

13-Baseline OIs A- No B-Yes

14-If Yes for q. No. 7(Mention).………………...................

15-OIs after ART initiation A-No B-Yes(mention)……………………

16-Baseline CD4 count (cells/mm3)

A-<200 B-200–350 C- 351–500 D-≥500

17-WHO Clinical stage A-stage-1 B-Stage-2 C-Stage-3 D-stage-4

18-Viral load (If Available)………………..

19-Baseline ART regimen (Mention all Drugs)…………………….

20-ART regimen changed A-No B-Yes

21-if Yes for q.no.20; Write the new drug Regimen…………………………………….

22-drug Adverse effect A-No B-yes (mention)………………………………….

23-Year of initiations A-≤2013 B- ≥2014

24-Adherence A-Poor (<95%) B- Good (≥95%)

Name and Signature of data collector…………………………………
